# The effects of genres on the development of multifaceted linguistic complexity in Chinese learners of German: A longitudinal corpus analysis

**DOI:** 10.1371/journal.pone.0326250

**Published:** 2025-06-16

**Authors:** Yushan Li, Yuan Li

**Affiliations:** 1 Institute of German Studies, School of International Studies, Zhejiang University, Hangzhou, China; The Chinese University of Hong Kong, HONG KONG

## Abstract

Genre-based research holds significant theoretical and practical importance in second language acquisition (SLA). While many L2 English writing studies have suggested argumentative writing was generally more challenging than narrative, whether this generalization applies to typologically different languages, such as German with its complex morphological and structural properties, requires further investigation. Specifically, genre effects on L2 writing development remain insufficiently understood for non-English languages, particularly regarding complexities beyond syntactic and lexical. This study examines how genre influences the development of morphological, lexical, syntactic, and cohesive complexity in elementary-intermediate Chinese learners of German over time. A longitudinal corpus of narrative and argumentative essays from 21 learners, who wrote both genres, was analyzed. The results show that genre effects were evident but not straightforward, with no genre consistently exhibiting greater complexity. Over time, learners predominantly exhibited nonlinear development patterns, suggesting the dynamic nature of SLA. Additionally, they exhibited decreasing lexical sophistication in both genres despite improvements in other complexity measures, indicating a “complexity trade-off” between linguistic subsystems. These findings contribute empirical evidence to the cross-linguistic comparisons in SLA research and suggest that curricula should incorporate diverse genres to develop multifaceted linguistic competence, with pedagogical approaches tailored to genre-specific complexity patterns and developmental trajectories.

## 1 Introduction

Traditionally, argumentative writing has been considered more complex than narrative writing due to its demands for logical reasoning and persuasive communication [1]. However, research on native German-speaking children has presented a different argument, showing greater writing fluency in argumentative texts compared to narratives [2]. This prompts an examination of whether the presumed complexities of argumentative writing apply across languages and learner contexts. Understanding these genre effects is crucial for both second language acquisition (SLA) research and pedagogical practice. Different genres serve distinct communicative purposes and thus are associated with unique linguistic features, with learners’ mastery of multiple genres playing a critical role in their overall communicative competence. Moreover, genre-based pedagogy supports writing development across languages including English and German [3,4]. Despite its recognized value, genre awareness in L2 German writing instruction in China remains limited, with an overemphasis on argumentative writing as the primary vehicle for language development and academic achievement. In light of these considerations, investigating how genre influences L2 writing development in non-English languages like German is essential.

In SLA research, linguistic complexity is widely employed to measure language development, as it captures the diversity and structural elaboration of learners’ language production [5]. Genre effects on writing development are often assessed through these complexity measures, which reflect various aspects of the linguistic system and encompass a wide range of language features [6]. Examining multiple facets of linguistic complexity allows for a more comprehensive understanding of L2 writing development, facilitating more nuanced cross-linguistic comparisons.

Argumentative texts in L2 English writing tend to exhibit greater syntactic complexity and lexical sophistication than narratives [1,7]. However, these findings often presuppose that “the relationship between complexity and development or proficiency is similar across languages” [8, p. 319], disregarding the specific challenges posed by typologically distinct languages like German, which features a rich morphological system and structural properties that are not fully captured in L2 English research. Moreover, while higher-proficiency learners are often the focus of research, the genre-specific writing features of lower-proficiency learners, who have been found to exhibit differences in their development relative to higher-proficiency learners [9], have been academically overlooked. Finally, although longitudinal studies on L2 German have provided insights into syntactic complexity development [10–12], few have examined genre effects over time or considered other key facets like morphology, which are important and challenging in learning German. To achieve an overall picture of linguistic complexity development across languages, proficiency levels, and genres, a more in-depth investigation into these overlooked areas is necessary. Thus, this study fills these gaps by investigating how genre influences the development of multifaceted linguistic complexity in the writing of elementary-intermediate Chinese learners of German. Focusing on genre effects and changes over time, this research provides nuanced perspectives on a less-studied learner group, target language, and linguistic facets. The findings contribute to both theoretical discussions in SLA and practical applications in language instruction, particularly in addressing the unique challenges of learning German.

The following section reviews literature on genre effects in the development of multifaceted linguistic complexity in L2 writing, with a particular emphasis on longitudinal studies involving German learners. Subsequently, an outline of the longitudinal study with 21 Chinese elementary-intermediate German learners is introduced, describing data collection of narrative and argumentative essays. Complexity measures, including morphological, lexical, syntactic, and cohesive, are further examined, along with statistical methods to analyze genre and time effects. The *Results* section presents significant findings, followed by an in-depth discussion to reveal genre-specific complexities as well as both shared and genre-specific developmental patterns in learners’ writing. Finally, we summarize main insights, acknowledge limitations, and suggest implications for language instruction and future research.

## 2 Literature review

### 2.1 Genre and linguistic complexity

Genres, shaped by social conventions, are characterized by specific linguistic features to fulfill distinct communicative purposes [13]. Research indicates that genres impact linguistic complexity in L2 writing [1,7,14]. Studies comparing narrative and non-narrative writing by the same L2 English learners have primarily focused on syntactic and lexical complexity, setting the stage for a deeper investigation.

[1] examined 37 advanced L2 English students’ narrative and argumentative essays over one semester, revealing significant genre effects, particularly in syntactic complexity. Argumentative essays had longer production units, including higher mean length of clauses (MLC), T-units (MLT), and sentences (MLS); more complex nominal structures; and greater coordination, such as more coordinate phrases per clause (CP/C), complex nominals per T-unit (CN/T), and verb phrases per T-unit (VP/T). Argumentative essays also displayed greater lexical sophistication (longer and less frequent words), while narratives had higher lexical richness and slightly longer text length.

Expanding on this research, [15] presented a more subtle perspective in their longitudinal study of 45 high-intermediate to advanced L2 English learners. They analyzed 270 essays written alternately in narrative and argumentative genres. Narrative writing demonstrated greater clausal complexity, with more dependent clauses per T-unit (DC/T) and a higher sentence coordination ratio (T/S), whereas argumentative essays exhibited greater phrasal complexity (higher MLC and CN/T) and higher lexical richness. As in prior studies, argumentative writing also demonstrated greater lexical sophistication.

Although genre effects have been widely studied in higher-proficiency L2 English learners, research on lower-proficiency learners remains limited. [9] investigated 360 argumentative and narrative essays by Chinese L2 English learners across three proficiency levels. Elementary learners exhibited genre effects only in clauses per T-unit (C/T), with narrative essays showing higher lexical sophistication and richness. In contrast, intermediate and advanced learners displayed clearer syntactic complexity differences between genres. Bi’s findings suggest that genre effects on linguistic complexity vary by proficiency, highlighting that conclusions from higher-proficiency learners may not apply to lower-level learners. Building on this, [16] examined morphological complexity in descriptive, narrative, and expository texts by elementary-level Iranian English learners. They found greater verb inflection variety (greater Morphological Complexity Index of verbs, MCI-Verb) in narrative texts, indicating that genre effects also occur in morphological complexity, especially among lower-proficiency learners.

Collectively, genre significantly influences linguistic complexity in L2 writing. In L2 English studies, argumentative essays tend to be more syntactically complex and lexically sophisticated, whereas narratives promote greater lexical richness, reflecting the distinct functional demands of each genre. It is worth noting that most research focuses on L2 English, primarily examining syntactic and lexical complexity in higher-proficiency learners.

### 2.2 Multifaceted linguistic complexity development in L2 German writing

In SLA research, complexity refers to both the number and variety of components and the relationships that connect them. Linguistic complexity can be evaluated across multiple domains, including lexis, morphology, syntax and cohesion, each with its own subdomains [6]. Such complexities are essential for benchmarking learner development alongside assessing learner proficiency and performance [5]. Recent studies on L2 German writing have uncovered substantial linguistic complexity development, advancing our understanding of changes in L2 German across time and complexity facets.

#### 2.2.1 Syntactic complexity development.

Using longitudinal data from the *Kansas Developmental Learner Corpus* (KANDEL) [17], Vyatkina and colleagues have extensively studied the syntactic complexity development in elementary- to intermediate-level L2 German learners. The writing tasks followed a clear curricular progression from personal narratives to personal accounts with reasoning elements, and then to argumentative and reflective writing, covering both narrative and argumentative genres.

[10] investigated lexical and syntactic complexity in the L2 German writing of beginning college-level learners over four semesters, noting significant increases in mean length of sentences (MLS) and in finite verb units per sentence (FV/S) (similar to clauses per sentence (C/S)). A shift from coordinating conjunctions to subordinating conjunctions was observed, with coordination negatively and subordination positively correlated with time. Lexical richness, measured by corrected type-token ratio (CTTR), also steadily improved, though words per FV-unit (W/FV) showed no clear increase, highlighting varied development across complexity measures. Building on this, [11] focused on two learners and revealed that despite individual differences, both learners also exhibited shared trends of increasing MLS, non-finite verb forms (NFV), and complex nominal structures. Both learners initially relied on coordinate clauses but progressed towards more advanced phrasal and clausal structures. [12] further explored syntactic modification across part-of-speech categories, finding increases in attributive adjectives and subordinate clauses, while cardinal numbers and predicative adjectives declined. Adverbs and prepositional phrases showed no clear trend. This suggested that as learners’ proficiency improves, they increasingly used more cognitively demanding structures like inflected adjectives and subordinate clauses, while simpler ones declined.

Collectively, these studies revealed key trends in syntactic complexity development among elementary to intermediate L2 German learners. There was a consistent increase in sentence length, subordination (including relative clauses), and complex nominal structures, while coordination and simpler structures steadily declined. However, the blending of genres in these studies complicates the interpretation of genre-specific development, as fluctuations in structures like coordination may be influenced by genre- and task-specific factors.

#### 2.2.2 Other linguistic complexity development.

Research on other facets of linguistic complexity development in L2 German writing is relatively scarce. Focusing on cohesive complexity, [18] analyzed longitudinal narrative texts from 30 Dutch learners with B2-C1 German proficiency (according to the Common European Framework of Reference for Languages (CEFR)) before and after an Erasmus semester. After the semester, learners demonstrated increased local noun and stem overlaps, more temporal connectors, higher lexical richness (TTR), and more hyponyms, enhancing cohesion and coherence. However, it is unclear if these results extend to other genres or lower-proficiency learners. In parallel, [19] explored connector use in 155 argumentative essays written by Chinese learners of German at A1 to B2 proficiency levels. They found that learners used fewer connectors than native speakers, with usage stabilizing from A1 to B1 and increasing significantly at B2. As a cross-sectional study, the results may have been influenced by individual differences, highlighting the need for longitudinal research to gain a clearer understanding of language development.

There are limited studies on morphological complexity in L2 German writing. However, German’s rich morphological system with its inflections poses significant challenges for learners. Learning inflectional morphology has been difficult, as it requires learners to understand both forms and functions, which often convey nuanced grammatical meanings not commonly shared across languages [20]. L2 German learners, especially from morphologically different L1s, often struggled with inflectional morphemes, with notable disparities between lower- and higher-proficiency learners [21]. Thus, studying morphological complexity development in elementary- to intermediate-level learners, particularly those with morphologically different native languages like Chinese, is essential for detailed insights into German language teaching. Regarding lexical complexity development, many of the aforementioned studies have consistently shown significant improvements in lexical richness across proficiency levels of German learners. However, lexical development involves not only vocabulary breadth but also depth of usage.

In summary, while research on L2 German writing has advanced in understanding certain facets, like syntactic development, it remains essential to account for the multiple facets of linguistic complexity. [22] found that multifaceted complexity, including text length, CTTR, dependent clauses, and temporal connectors, better classified proficiency levels of L2 German than homogeneous measures. Furthermore, [23] showed that features like genitive case morphological inflection, word frequency, noun phrase ratio, and relative clause ratio were related to German writing quality, highlighting the need to study multifaceted linguistic complexity in L2 German.

## 3 The present study

This study aims to examine the development of morphological, lexical, syntactic, and cohesive complexity in narrative and argumentative writing by L2 German learners at the elementary-intermediate proficiency level over time. As discussed in the literature review, genre effects on L2 writing development remain unclear for non-English languages, particularly concerning lower-proficiency learners and for complexities beyond syntactic and lexical. To address this, we pose two research questions:

RQ 1: In which linguistic complexity indicators, and to what extent, does genre affect multifaceted linguistic complexity in the writing of elementary-intermediate Chinese learners of German?

RQ 2: How does multifaceted linguistic complexity develop over time in L2 German narrative and argumentative writing?

### 3.1 Data and participants

The participants comprised 21 native Chinese speakers majoring in German at a university in China. All participants were in the same academic year and had no prior experience learning German. They followed a standardized German program with a shared curriculum and teaching team, completing 192 intensive German lesson hours in each of the first two semesters and 160 in the third and fourth. All participants were admitted to the same major through the National College Entrance Examination (Gaokao), suggesting similar general learning capacity and prior language learning potential. The participants formed a homogeneous group in terms of language background, instructional input, and initial language proficiency. According to the Goethe-Institut [24], a prominent German cultural organization promoting the German language and culture, learners who complete 600–750 hours of instruction typically reach the B2 level of the CEFR, suggesting that participants would be expected to reach B2 proficiency by the end of the fourth semester. Throughout this study, they were at an elementary-intermediate proficiency level, with exposure to German primarily limited to classroom interactions and teaching materials.

This study focused on narrative and argumentative writing, selected for their communicative differences and comparability with L2 English studies. The topics for narrative writing were *My Family* and *An Unforgettable Experience*, and for argumentative were *Should Mobile Phones Be Allowed in Classrooms?* and *Pursue Graduate Studies or Start Working?* To minimize prompt influence, all prompts were provided in Chinese. Essays were handwritten in class within 30 minutes, without access to external resources, and were later digitized through a careful manual transcription process. Each essay was typed into electronic format by a trained research assistant who preserved all original errors in the learners’ writing. A second research assistant then independently verified each transcription against the original handwritten document to identify and correct any transcription errors. This two-step verification process was implemented to minimize the introduction of transcription errors while preserving the authentic features of learners’ writing for subsequent linguistic analysis.

Essays were collected across the first four semesters (Table 1). Narrative essays were collected at the end of the first (December 14, 2022), second (June 7, 2023), and third (December 6, 2023) semesters. Argumentative essays were collected at the end of the second (June 7, 2023), third (December 6, 2023), and fourth (June 5, 2024) semesters. During the second and third semesters, participants wrote both a narrative and an argumentative essay at each data collection point.

**Table 1:** Corpus composition and analytical usage across four semesters.

Collection time (Semester)	Argumentative		Narrative		Analytical usage
	Texts	Average tokens	Texts	Average tokens	
1	0	0	21	136.48	Longitudinal (Narrative only)
2	21	107.95	21	134.95	Cross-genre comparison & Longitudinal analysis
3	21	134.76	21	156.95	Cross-genre comparison & Longitudinal analysis
4	21	160.33	0	0	Longitudinal (Argumentative only)
Total	63		63		Total corpus: 126 essays

Shaded cells indicate the overlapping period where both genres were collected simultaneously, providing data for direct cross-genre comparisons. All available time points for each genre were used for longitudinal analysis.

The staggered data collection was due to curricular sequencing and proficiency limitations. Ideally, data for both genres would have been collected at all four time points to allow for direct comparisons across genres while controlling for proficiency and other variables. However, at the end of the first semester, participants’ proficiency was insufficient for argumentative essays, so only narrative essays were collected. By the fourth semester, the curriculum focused primarily on argumentative writing, making it impractical to collect narrative essays in class. Despite this, the overlapping period (the second to the third semester) enabled a direct cross-genre comparison. The analysis will follow two approaches: (1) For genre effects, only data from the overlapping period will be used; (2) For longitudinal development, all three time points for each genre will be analyzed.

### 3.2 Measurement and indicators

This study employed the *Common Text Analysis Platform* (CTAP) [25] to analyze the morphological, lexical, syntactic, and cohesive complexity in L2 German writing. Previous studies [18,22] have validated CTAP’s reliability. Below are the specific indicators used, with a summary provided in S1 Table.

#### 3.2.1 Morphological complexity.

Five indicators focusing on verbs and case inflections were selected to measure morphological complexity. Specifically, Morphological Complexity Index of verbs (MCI-Verb) assesses the “average inflectional diversity for [verbs] in a text” [20, p. 103]. Since verb inflection occurs across languages – from “poor” ones like English to richer ones like German – MCI-Verb enables meaningful cross-linguistic comparisons and remains unaffected by text length, making it an effective measure of learners’ morphological development [26].

German’s four grammatical cases – nominative, accusative, dative, and genitive – each serves characteristic grammatical functions, which are signaled through a system of case inflections. Typically, the nominative marks subjects, the accusative marks direct objects, the dative marks indirect objects, and the genitive indicates possession [27]. This inflectional system and the functional distinctions of cases present significant learning challenges for Chinese learners, whose L1 lacks comparable morphological marking. Thus, we measured nominative, accusative, dative, and genitive case inflections per token. In sum, these five indicators offer a comprehensive assessment of morphological development in L2 German learners.

#### 3.2.2 Lexical complexity.

Lexical complexity was measured through lexical richness, density, and sophistication [28]. Lexical richness, traditionally assessed by TTR, refers to the number of different words in a learner’s vocabulary [29]. Since TTR is sensitive to text length, this study used its variant CTTR [30]. Lexical density measures the proportion of lexical words (e.g., nouns, adjectives) to total words [31]. Lexical sophistication is defined as the use of advanced, low-frequency words [28], which are identified in this study based on word frequency and age of acquisition. Specifically, we employed two indicators: the average word frequency (based on the *Google Books Frequency List)* and the average age of active use of words (This indicator is calculated by determining the mean age at which native German-speaking children actively use these words in their writing, as documented in the *Karlsruhe Children’s Text Corpus,* which comprises texts written by native German-speaking children). While this age-related indicator is not identical to the “age of acquisition”, we assume that words actively used earlier by children are easier for L2 learners to process. Together, these four indicators provide a detailed insight into the lexical complexity in L2 German writing.

#### 3.2.3 Syntactic complexity.

We initially considered 14 syntactic complexity measures proposed by [7], including production unit length (e.g., mean length of clauses (MLC)), sentence complexity (clauses per sentence (C/S)), subordination (e.g., dependent clauses per T-unit (DC/T)), coordination (e.g., coordinate phrases per clause (CP/C)), and particular structures (e.g., complex nominals per T-unit (CN/T)), many of which have been applied to L2 German [11,32,33]. However, broad measures like T-unit length may not fully capture specific syntactic features [34]. To address this, [12] introduced finer-grained syntactic complexity indicators for L2 German writing, focusing on sentential, clausal, and phrasal modifications. These were operationally defined as “specific clause types and phrases headed by [six] particular POS” (p. 32), including adjectives, cardinal numbers, adverbs, prepositional phrases, subordinating conjunctions, and relative pronouns. This study adopted these syntactic modifiers as key indicators, incorporating a total of 20 syntactic complexity indicators. To avoid redundancy and multicollinearity [35], we conducted pairwise correlation analyses. Eight measures were excluded due to high correlations (Pearson’s *r* > 0.70), leaving 12 final indicators.

#### 3.2.4 Cohesive complexity.

Cohesion refers to linguistic elements that connect ideas within a text [36] and can be divided into local and overall (or global) cohesion [37]. Local cohesion involves devices like connectors that explicitly establish connections in discourse, along with the proportion of adjacent sentence pairs that share one or more arguments, stems, or lexical items, thus implicitly creating cohesion between sentences. Global cohesion relates to connections across larger units, such as paragraphs. For example, global argument overlap measures the proportion of sentence pairs within a text that share the same argument. Cohesive devices serve to mark logical relationships between text segments, facilitating reader comprehension. The use of these devices is essential for constructing coherent texts. It has been shown that learners’ development of cohesion skills generally parallels their overall writing proficiency development [37].

Based on these distinctions, we selected ten cohesion measures. First, for connectives, we calculated the ratio of all connectors per token, using Breindl et al.’s list [38]. Following [18], we also included temporal connectors per token, as these were found to be significant measures of development in L2 German. Next, we evaluated the remaining eight indicators, measuring the overlaps of nouns, arguments, content words, and lemmas at both local and global levels. However, indicators with over 80% zero values, namely, local overlaps of nouns, content words, and lemmas, were excluded. After conducting pairwise correlation analyses, three more were removed due to high correlations (Pearson’s *r* > 0.70), leaving four indicators: all connectors per token, local and global argument overlaps, and global lemma overlap.

Text length, measured by the total number of tokens, was also selected as a general measure. Although the task in this study was time-limited, there was no word limit, which allows longer text to potentially reflect greater overall language proficiency. Previous research [39,40] has shown that text length reliably indicates L2 writing quality and development. Additionally, it correlated positively with cohesive complexity in L2 German writing [18].

In summary, this study employed 26 indicators to measure multifaceted linguistic complexity in L2 German writing, covering morphological, lexical, syntactic, and cohesive complexity measures.

### 3.3 Data analysis

We used CTAP to compute values for each complexity indicator in the essays, followed by addressing multicollinearity. Prior to further analysis, we tested the data for normality using Shapiro-Wilk tests, which showed that most variables did not meet normality assumptions. Given this and the small sample size, non-parametric methods were employed. To address the first research question, we conducted Wilcoxon signed-rank tests with Genre as a within-subject variable, comparing the multifaceted linguistic complexity of narrative and argumentative texts written by the 21 participants at the same time point during the overlapping observation period to identify significant differences between the two genres.

For the second research question, we performed Friedman tests with Time as the within-subject variable to examine changes in multifaceted linguistic complexity for both narrative and argumentative genres over time. For indicators showing significant results in the Friedman tests, we followed up with pairwise comparisons using Wilcoxon signed-rank tests to identify specific developmental patterns between semesters.

To control for Type I error, all *p*-values were adjusted using the Benjamini-Hochberg correction (hereafter referred to as BH-adjusted *p*), ensuring statistical rigor across the complexity indicators.

## 4 Results

### 4.1 Genre effects on linguistic complexity

The Wilcoxon signed-rank test assessed multifaceted linguistic complexity in elementary-intermediate German learners’ narrative and argumentative essays. Overall, learners demonstrated greater morphological complexity and produced longer texts in narratives (*Z* = −3.240, BH-adjusted *p* = 0.003, *r* = −0.500), whereas argumentative writing displayed higher lexical, syntactic, and cohesive complexity (Table 2).

**Table 2:** Wilcoxon signed-rank test results on genre effects in L2 German writing.

Indicator	Complexity	Narrative Med (Q1 - Q3)	Argumentative Med (Q1 - Q3)	*Z*	*p* (BH)	*r*	Complex
Text length	Global	137.000 (107.750 - 157.750)	114.500 (98.500 - 135.500)	−3.240	0.003	−0.500	N > A
MCI-Verb	Morphological	4.000 (4.000 - 4.500)	2.500 (2.000 - 3.625)	−4.537	< 0.001	−0.700	N > A
Inflection (nominative)	Morphological	0.591 (0.540 - 0.641)	0.611 (0.563 - 0.647)	−0.781	0.488	−0.121	
Inflection (genitive)	Morphological	0.030 (0.020 - 0.044)	0.026 (0.013 - 0.041)	−1.302	0.251	−0.201	
Inflection (dative)	Morphological	0.218 (0.198 - 0.248)	0.162 (0.128 - 0.229)	−3.170	0.004	−0.489	N > A
Inflection (accusative)	Morphological	0.143 (0.109 - 0.174)	0.208 (0.181 - 0.248)	−4.545	< 0.001	−0.701	A > N
Lexical richness (CTTR)	Lexical	5.531 (5.245 - 5.947)	5.076 (4.707 - 5.357)	−4.858	< 0.001	−0.750	N > A
Lexical density	Lexical	0.459 (0.436 - 0.483)	0.516 (0.491 - 0.536)	−4.895	< 0.001	−0.755	A > N
Word frequency	Lexical	3688.580 (3230.948 - 4320.388)	3754.090 (3091.340 - 4928.153)	−1.844	0.094	−0.285	
Mean age of active use	Lexical	10.657 (10.581 - 10.759)	10.948 (10.854 - 11.116)	−5.583	< 0.001	−0.861	A > N
Mean length of clause	Syntactic	6.667 (6.078 - 7.355)	8.067 (6.957 - 8.844)	−3.807	0.001	−0.587	A > N
Sentence coordination ratio	Syntactic	0.946 (0.888 - 1.000)	1.000 (0.894 - 1.000)	−0.754	0.488	−0.116	
Coordinate phrases per T-unit	Syntactic	0.074 (0.000 - 0.134)	0.174 (0.000 - 0.276)	−3.094	0.004	−0.477	A > N
Sentence complexity ratio	Syntactic	1.341 (1.254 - 1.628)	1.364 (1.229 - 1.579)	−0.227	0.821	−0.035	
Dependent clauses per T-unit	Syntactic	0.261 (0.174 - 0.404)	0.369 (0.229 - 0.560)	−3.145	0.004	−0.485	A > N
Complex nominals per T-unit	Syntactic	0.325 (0.219 - 0.530)	0.515 (0.333 - 0.628)	−3.339	0.003	−0.515	A > N
Subordinating conjunction density	Syntactic	0.028 (0.019 - 0.033)	0.029 (0.020 - 0.044)	−1.119	0.326	−0.173	
Relative pronoun density	Syntactic	0.000 (0.000 - 0.000)	0.000 (0.000 - 0.001)	−0.569	0.592	−0.088	
Adjective density	Syntactic	0.070 (0.058 - 0.086)	0.064 (0.044 - 0.087)	−1.882	0.092	−0.290	
Cardinal number density	Syntactic	0.007 (0.000 - 0.009)	0.000 (0.000 - 0.000)	−3.116	0.004	−0.481	N > A
Adverb density	Syntactic	0.065 (0.050 - 0.082)	0.055 (0.042 - 0.079)	−1.357	0.239	−0.209	
Prepositional phrases per T-unit	Syntactic	0.629 (0.484 - 0.828)	0.798 (0.529 - 1.216)	−2.235	0.041	−0.345	A > N
Local argument overlap	Cohesive	0.077 (0.000 - 0.098)	0.000 (0.000 - 0.111)	−0.872	0.453	−0.135	
Global argument overlap	Cohesive	1.000 (0.534 - 1.636)	0.641 (0.421 - 1.000)	−2.507	0.021	−0.387	N > A
All connectors per token	Cohesive	0.124 (0.072 - 0.158)	0.187 (0.101 - 0.250)	−2.572	0.019	−0.397	A > N
Global lemma overlap	Cohesive	0.093 (0.000 - 0.182)	0.317 (0.108 - 0.510)	−4.274	< 0.001	−0.659	A > N

Median (Med) values are shown with interquartile ranges (Q1 - Q3). *Z* denotes the Wilcoxon signed-rank test statistic, with corresponding *p*-values. *p* (BH) represents *p*-values adjusted using the Benjamini-Hochberg method. “A > N” indicates that argumentative writing was more complex than narrative writing for the given indicator, while “N > A” indicates the reverse.

In narratives, learners produced significantly higher verb morphological complexity (*Z* = −4.537, BH-adjusted *p* < 0.001, *r* = −0.700), more frequent dative case inflections (*Z* = −3.170, BH-adjusted *p* = 0.004, *r* = −0.489), and higher lexical richness (*Z* = −4.858, BH-adjusted *p* < 0.001, *r* = −0.750). Additionally, they used more cardinal numbers (*Z* = −3.116, BH-adjusted *p* = 0.004, *r* = −0.481) and exhibited higher global argument overlap (*Z* = −2.507, BH-adjusted *p* = 0.021, *r* = −0.387).

Argumentative essays were characterized by more accusative case inflection usage (*Z* = −4.545, BH-adjusted *p* < 0.001, *r* = −0.701), higher lexical density (*Z* = −4.895, BH-adjusted *p* < 0.001, *r* = −0.755), and a higher mean age of active use of words (*Z* = −5.583, BH-adjusted *p* < 0.001, *r* = −0.861). Additionally, argumentative texts had longer clauses (*Z* = −3.807, BH-adjusted *p* = 0.001, *r* = −0.587), more dependent clauses, coordinate phrases, complex nominals and prepositional phrases (*Z* = −3.145, −3.094, −3.339, −2.235, BH-adjusted *p* = 0.004, 0.004, 0.003, 0.041, *r* = −0.485, −0.477, −0.515, −0.345, respectively), more connectors overall (*Z* = −2.572, BH-adjusted *p* = 0.019, *r* = −0.397), and higher global lemma overlap (*Z* = −4.274, BH-adjusted *p* < 0.001, *r* = −0.659).

**Fig 1:**
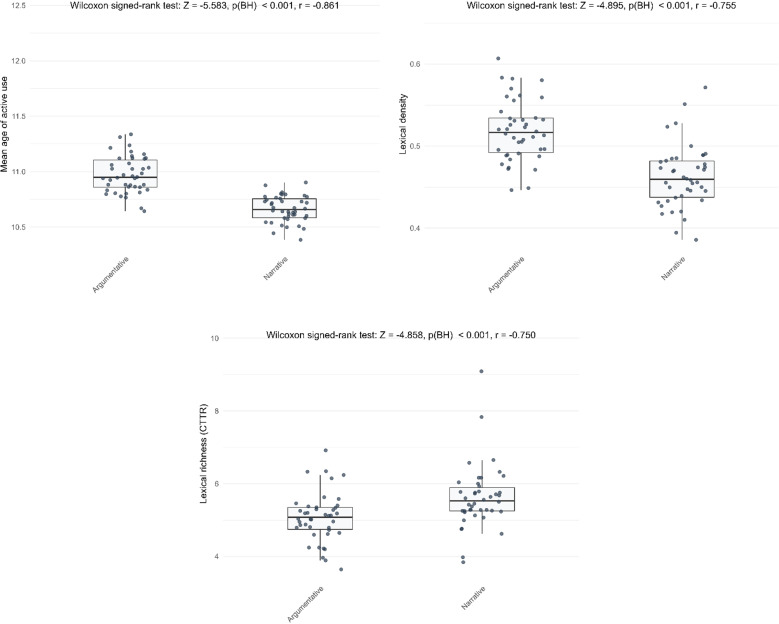
The three indicators with the largest effect sizes in genre differences.

The largest genre differences were observed in lexical complexity, with the greatest effect sizes in mean age of active use, lexical density, and richness (Fig 1). Overall, during the early stages of learning German, learners produced richer vocabulary in narrative writing, whereas lexical sophistication and density were higher in argumentative writing.

### 4.2 Linguistic complexity development in different genres

To track the developmental trajectories of linguistic complexity in L2 German writing across genres, separate longitudinal analyses were conducted on argumentative essays (from the second to the fourth semester) and narrative essays (from the first to the third semester) written by the same cohort of 21 learners. For each genre, Friedman tests with Benjamini-Hochberg correction (*α* = 0.05) were applied to all 26 complexity indicators across the respective three-semester observation windows. Subsequently, for those indicators that showed significant changes, we performed pairwise Wilcoxon signed-rank tests with adjusted p-values to identify specific semester-to-semester developmental patterns.

#### 4.2.1 Development in argumentative writing.

The longitudinal analysis of L2 German argumentative writing from the second to the fourth semester (S2 to S4) indicated that 18 out of the 26 complexity indicators changed significantly over time (Friedman tests with Benjamini-Hochberg adjusted *p* < 0.05). Further pairwise comparisons (Wilcoxon signed-rank tests) identified three developmental patterns among these indicators: continuous linear development, nonlinear stage-wise development, and cumulative development (S2 Table).

**Fig 2:**
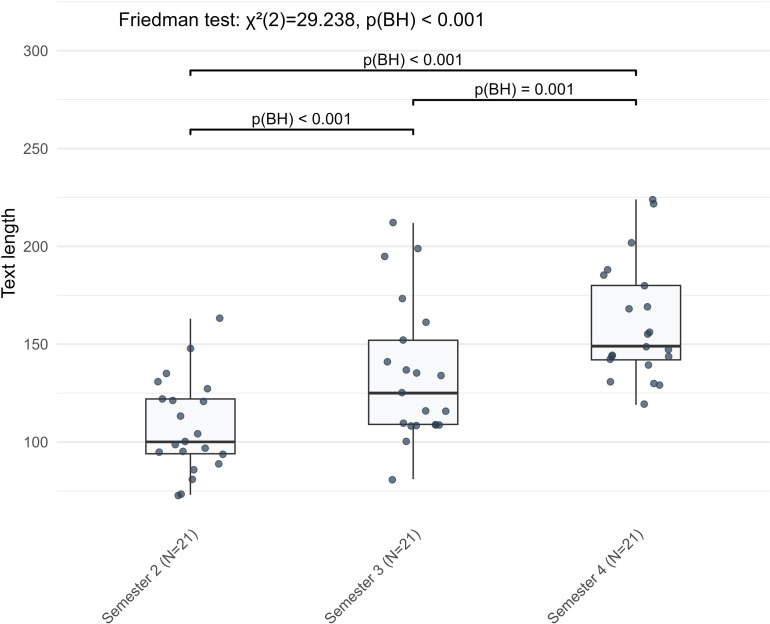
The linear development pattern of text length in L2 German argumentative writing across three semesters.

**Fig 3:**
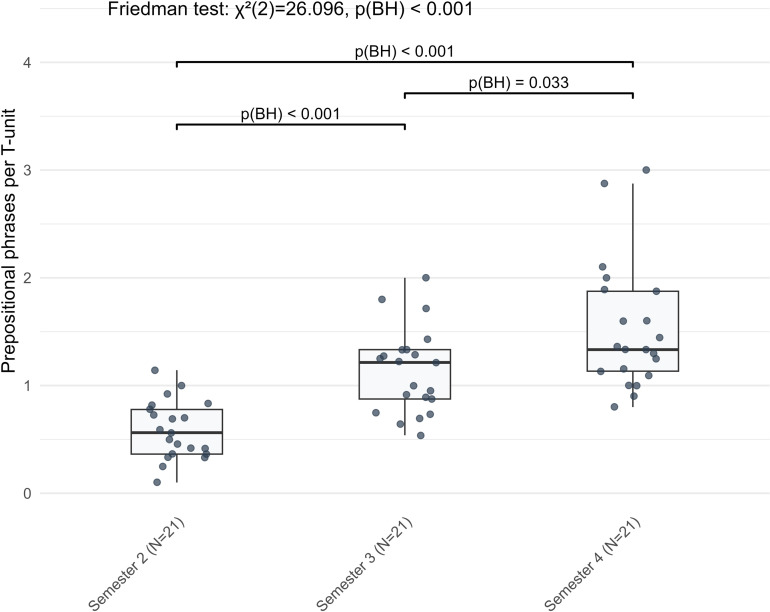
The linear development pattern of prepositional phrases per T-unit in L2 German argumentative writing across three semesters.

The continuous linear development pattern was observed in six complexity indicators, all exhibiting consistent semester-by-semester growth. Among them, text length showed the most substantial increase (*χ²* = 29.238, BH-adjusted *p* < 0.001), with sustained significant growth across both observation periods (S2 to S3: *Z* = −3.772, BH-adjusted *p* < 0.001; S3 to S4: *Z* = −3.303, BH-adjusted *p* < 0.001) (Fig 2). Similarly, learners used the dative case inflections more frequently over time (*χ²* = 18.952, BH-adjusted *p* < 0.001; S2 to S3: *Z* = −2.833, BH-adjusted *p* = 0.007; S3 to S4: *Z* = −3.597, BH-adjusted *p* < 0.001) and employed a more varied vocabulary (*χ²* = 17.238, BH-adjusted *p* < 0.001, S2 to S3: *Z* = −2.868, BH-adjusted *p* = 0.006, S3 to S4: *Z* = −2.172, BH-adjusted *p* = 0.042). In terms of syntactic complexity, both clause length (*χ²* = 18.667, BH-adjusted *p* < 0.001, S2 to S3: *Z* = −2.520, BH-adjusted *p* = 0.018, S3 to S4: *Z* = −2.416, BH-adjusted *p* = 0.023) and the frequency of prepositional phrases (*χ²* = 26.096, BH-adjusted *p* < 0.001, S2 to S3: *Z* = −3.771, BH-adjusted *p* < 0.001, S3 to S4: *Z* = −2.260, BH-adjusted *p* = 0.033) (Fig 3) increased progressively across semesters. Within cohesive complexity, global lemma overlap, also increased steadily from S2 to S4 (*χ²* = 25.268, BH-adjusted *p* < 0.001, S2 to S3: *Z* = −3.582, BH-adjusted *p* < 0.001, S3 to S4: *Z* = −2.103, BH-adjusted *p* = 0.050, exact BH-adjusted *p* = 0.0498). Overall, six linguistic complexity indicators across morphological, lexical, syntactic and cohesive complexities showed linear development in L2 German argumentative writing from the second to the fourth semester.

**Fig 4:**
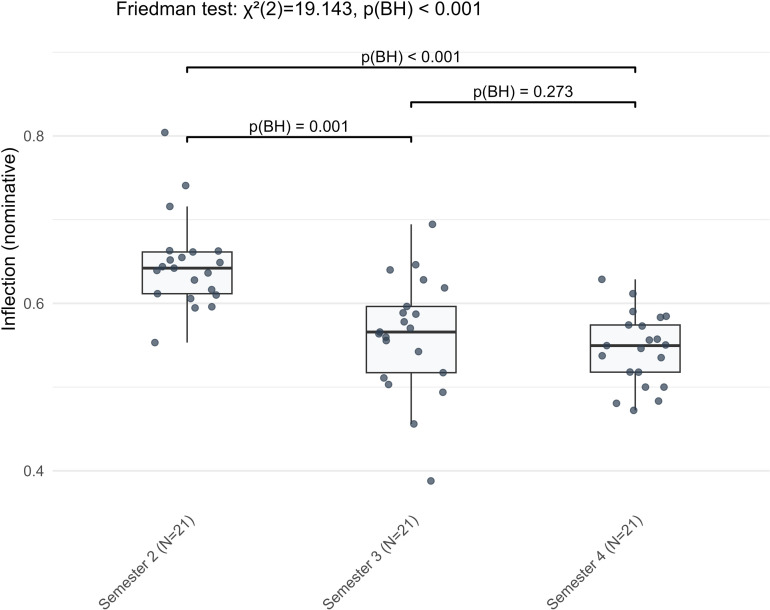
The nonlinear stage-wise development pattern of inflection (nominative) in L2 German argumentative writing across three semesters.

**Fig 5:**
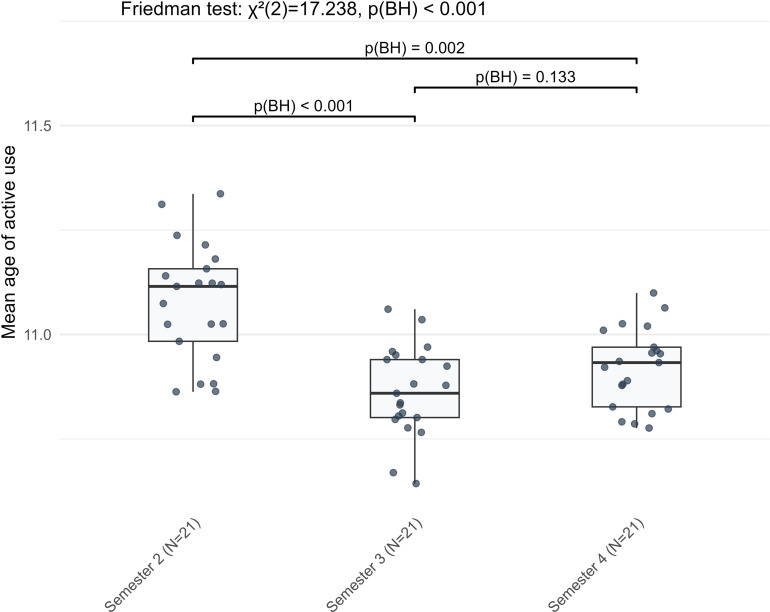
The nonlinear stage-wise development pattern of mean age of active use in L2 German argumentative writing across three semesters.

Ten indicators exhibited a nonlinear stage-wise development, characterized by a significant change at one interval (either S2 to S3 or S3 to S4) but not at both. Most indicators differed significantly between S2 and S3, followed by limited further change from S3 to S4. However, a few indicators displayed the opposite trend, with significant variation occurring only between S3 and S4. Morphologically, learners used fewer nominative case inflections in S3 than in S2 (*Z* = −3.319, BH-adjusted *p* = 0.001) (Fig 4), while genitive case inflections became more frequent during the same period (*Z* = −3.632, BH-adjusted *p* < 0.001). Neither indicator showed further significant changes by S4. In contrast, accusative case inflections maintained similar frequencies between S2 and S3, but decreased by S4 (*Z* = −2.728, BH-adjusted *p* = 0.009). Two lexical sophistication indicators varied inversely between S2 and S3: Learners tended to write words of higher frequency (*Z* = −3.771, BH-adjusted *p* < 0.001), while the mean age of active use of words declined (*Z* = −3.806, BH-adjusted *p* < 0.001) (Fig 5). Both indicators remained largely unchanged from S3 to S4. Syntactically, from S2 to S3, learners employed more complex nominals (*Z* = −2.763, BH-adjusted *p* = 0.008) and used relative pronouns and cardinal numbers more often (*Z* = −2.666, −2.371, BH-adjusted *p* = 0.008, 0.025, respectively). Meanwhile, their use of adverbs decreased significantly during this period (*Z* = −2.902, BH-adjusted *p* = 0.005). Notably, dependent clauses per T-unit did not change significantly from S2 to S3 but increased in S4 (*Z* = −2.312, BH-adjusted *p* = 0.029). Taken together, these ten indicators displayed diverse overall trends. Learners reduced their use of nominative and accusative cases while increasing genitive case usage. Lexical sophistication shifted toward more frequent words and those used earlier by native German-speaking children. Syntactic development was evident in the increased use of complex structures, namely, dependent clauses, complex nominals, and relative pronouns, alongside reduced use of cardinal numbers and adverbs.

**Fig 6:**
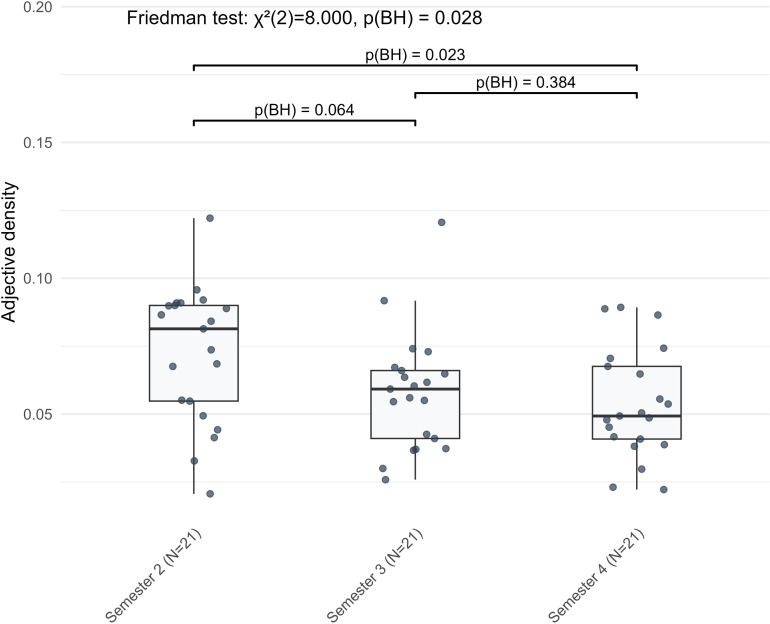
The cumulative development pattern of adjective density in L2 German argumentative writing across three semesters.

**Fig 7:**
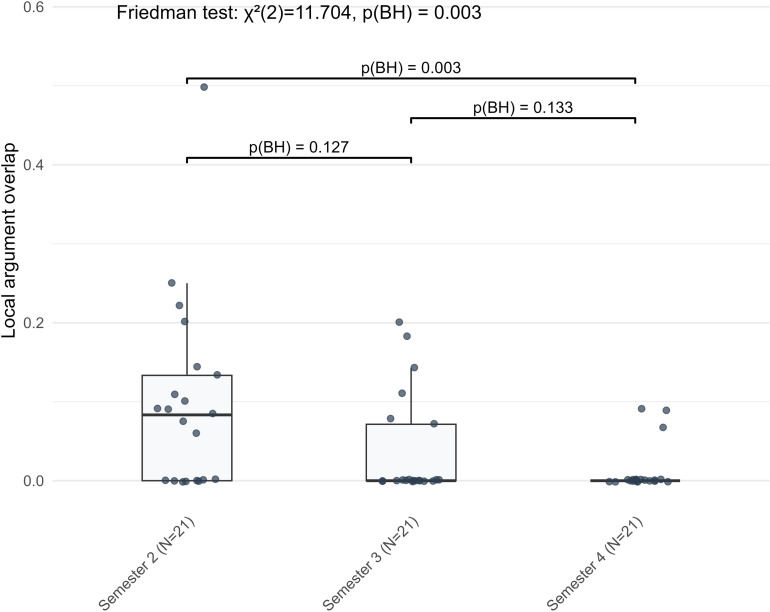
The cumulative development pattern of local argument overlap in L2 German argumentative writing across three semesters.

A cumulative development pattern was evident in two complexity indicators, both of which showed reductions from S2 to S4 without significant differences between adjacent semesters. Adjective density revealed an overall decline (*Z* = −2.416, BH-adjusted *p* = 0.023), but none of the successive comparisons reached statistical significance (Fig 6). A similar pattern was found for local argument overlap, which also underwent a discontinuous decline across the observation period (*Z* = −2.970, BH-adjusted *p* = 0.003), while adjacent comparisons remained non-significant (Fig 7). Both indicators showed changes that became statistically evident only across the full three-semester span, marking a non-sequential pattern of development distinct from the other two.

#### 4.2.2 Development in narrative writing.

A parallel set of longitudinal analyses was conducted on L2 German narrative writing, spanning from the first to the third semester (S1 to S3). Of the 26 complexity indicators examined, 16 showed significant changes over time, as determined by Friedman tests with Benjamini-Hochberg correction (α = 0.05). Follow-up Wilcoxon signed-rank tests identified two developmental patterns: bidirectional fluctuation in coordinate phrase usage and nonlinear stage-wise development in fifteen indicators (S3 Table).

**Fig 8:**
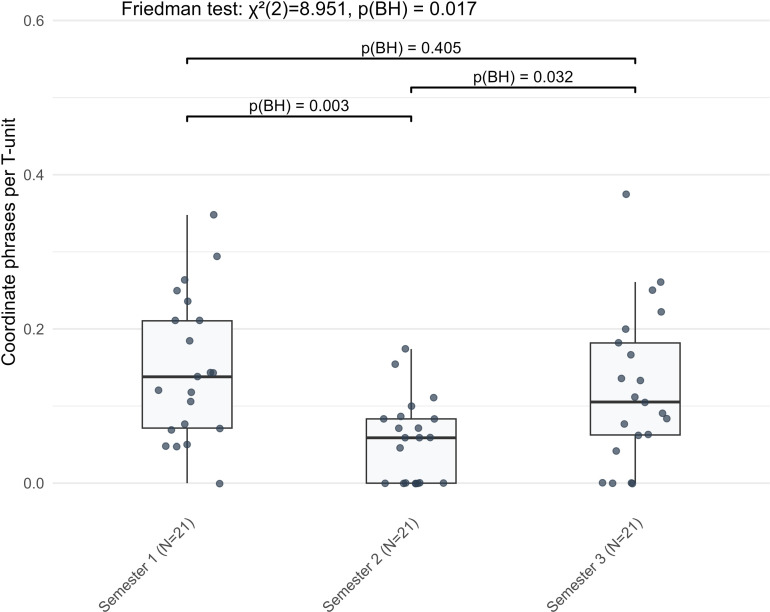
The U-shaped pattern of coordinate phrases per T-unit in L2 German narrative writing across three semesters.

The only bidirectional trend, a U-shaped trajectory, emerged in coordinate phrases per T-unit (Fig 8). Learners used fewer coordinate phrases from S1 (median = 0.138) to S2 (median = 0.059, *Z* = −3.024, BH-adjusted *p* = 0.003). By S3, their usage significantly increased (median = 0.105, *Z* = −2.296, BH-adjusted *p* = 0.032). This pattern of decline and subsequent ascent resulted in a statistically significant overall variation across the semesters (*χ²* = 8.951, BH-adjusted *p* = 0.017), although the net change between S1 and S3 was not statistically significant. This trend was distinct from the unidirectional patterns observed in other indicators.

**Fig 9:**
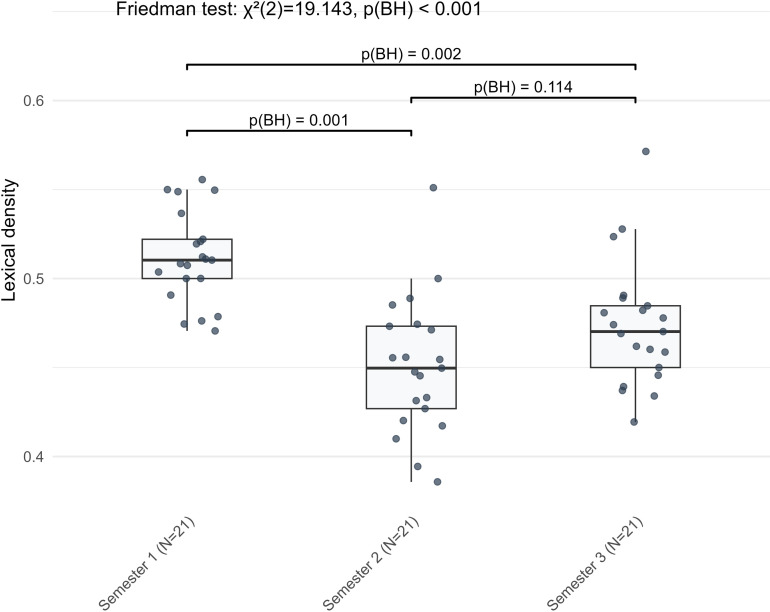
The nonlinear stage-wise development of lexical density in L2 German narrative writing across three semesters.

**Fig 10:**
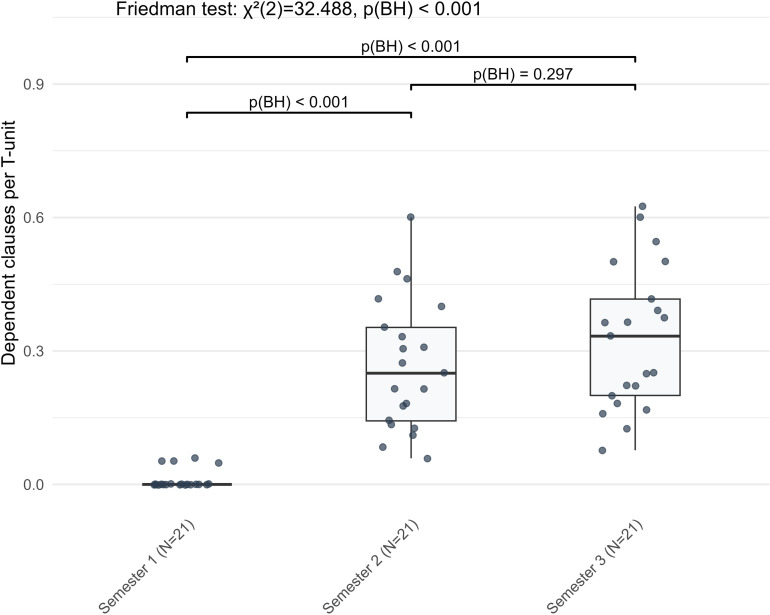
The nonlinear stage-wise development of dependent clauses per T-unit in L2 German narrative writing across three semesters.

Nonlinear stage-wise development characterized fifteen complexity indicators, with changes unfolding along two temporal trajectories. Thirteen indicators varied notably between S1 and S2, followed by statistical stability in S3, while two lexical sophistication indicators changed significantly only between S2 and S3. Within morphological complexity, both verb inflection diversity and nominative case inflections rose from S1 to S2 (*Z* = −3.010, −3.180, BH-adjusted *p* = 0.003, 0.002, respectively), with no further change in S3. Conversely, genitive and accusative case inflections decreased from S1 to S2 (*Z* = −3.354, −3.250, BH-adjusted *p* = 0.001, 0.002 respectively), followed by statistical stability. Regarding lexical complexity, lexical richness and lexical density presented diverging trends. While CTTR increased significantly from S1 to S2 (*Z* = −3.389, BH-adjusted *p* = 0.001), lexical density declined (S1 to S2: *Z* = −3.424, BH-adjusted *p* = 0.001) (Fig 9). Syntactically, learners constructed longer clauses, produced more clauses overall, and made more frequent use of dependent clauses, subordinating conjunctions, and prepositional phrases from S1 to S2, with no further changes in S3. This was statistically supported by increases in mean clause length, sentence complexity ratio, dependent clauses per T-unit (Fig 10), subordinating conjunction density, and prepositional phrases per T-unit (PP/T) (*Z* = −3.702, −3.146, −4.015, −4.015, −3.667, respectively; all BH-adjusted *p* < 0.001, except for sentence complexity ratio, where BH-adjusted *p* = 0.002). Cardinal number density decreased from S1 to S2 (Z = −3.667, BH-adjusted p < 0.001) and likewise remained unchanged afterward. Regarding cohesive complexity, global lemma overlap exhibited a significant decrease from S1 to S2 (Z = −3.215, BH-adjusted p = 0.002), whereas neither the comparison between S2 and S3 nor that between S1 and S3 yielded statistically detectable differences.

**Fig 11:**
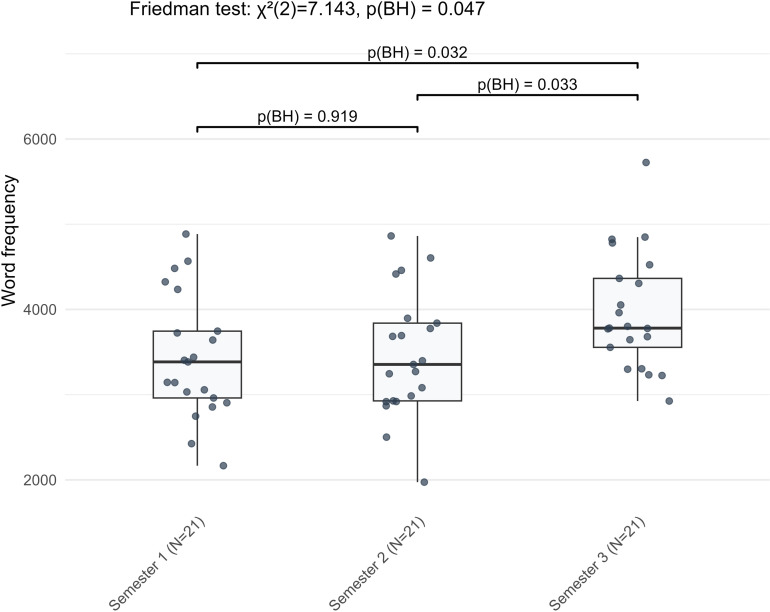
The nonlinear stage-wise development of word frequency in L2 German narrative writing across three semesters.

**Fig 12:**
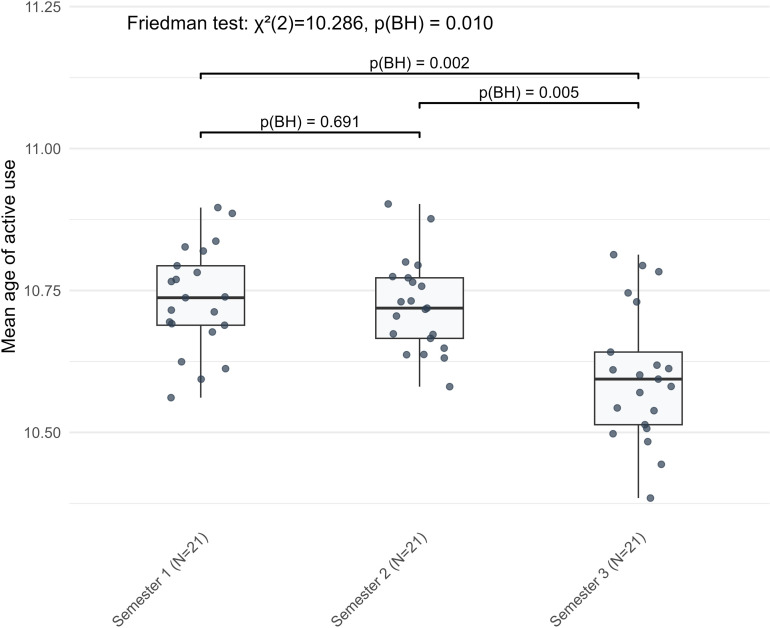
The nonlinear stage-wise development of mean age of active use in L2 German narrative writing across three semesters.

While the majority of indicators varied between S1 and S2, two lexical sophistication indicators showed significant changes only between S2 and S3. Word frequency rose (*Z* = −2.277, BH-adjusted *p* = 0.033) (Fig 11), and the mean age of active use declined (*Z* = −2.868, BH-adjusted *p* = 0.005) (Fig 12). Neither indicator differed significantly between S1 and S2. Taken as a whole, these fifteen indicators followed several broad trends. Learners used more diverse verb inflections and relied more on the nominative case, while genitive and accusative cases declined. Lexical richness and word frequency increased, whereas lexical density and the mean age of active word use decreased. Syntactic complexity rose through longer and more numerous clauses, with more dependent clauses and subordinating conjunctions, alongside greater use of prepositional phrases. In contrast, the use of cardinal numbers showed a general decline, and global lemma overlap also decreased.

Overall, the longitudinal analyses revealed distinct genre-specific trajectories in the development of multifaceted linguistic complexity among elementary-intermediate L2 German learners. In argumentative writing (S2 to S4), 18 of the 26 indicators showed statistically significant differences, following three primary patterns: continuous linear development, nonlinear stage-wise development, and cumulative development, with the majority of changes occurring between the second and third semesters. In narrative writing (S1 to S3), two patterns were identified: a U-shaped fluctuation in coordinate phrase usage, and nonlinear stage-wise development, with most significant changes observed between the first and second semesters. Across both genres, nonlinear trajectories dominated. A closer comparison of the overlapping observation window (S2 to S3) revealed broader and more multifaceted complexity development in argumentative writing, where 14 indicators exhibited significant variation. In contrast, narrative writing displayed more limited development during this same period, with changes confined to three indicators related to lexical and syntactic complexities. These findings highlight both shared and genre-specific patterns in L2 German writing development and provide a foundation for the discussion that follows.

## 5 Discussion

### 5.1 Genre effects on linguistic complexity

The first research question examined how genre impacts linguistic complexity. Our findings revealed that genre effects were distinct but not straightforward, with no genre generally being more complex across all measures. Broadly speaking, narrative writing showed greater morphological complexity and longer text length, while argumentative writing was characterized by higher lexical density and sophistication, greater syntactic complexity, and stronger cohesion. However, both genres displayed unique complexities across different linguistic facets, without any genre surpassing the other.

In narrative writing, learners frequently used verbs requiring dative case arguments, which often served to recount events and describe character interactions. Additionally, they employed prepositions governing dative cases to express circumstantial details such as time, place, and mode. For instance, verbs like *helfen* (means *help*) or *schenken* (means *give as a present*) take dative objects as beneficiaries, while *schmecken* (means *taste*) and *danken* (means *thank*) use datives to mark the target of emotions or attitudes. Examples of such prepositions include *mit* (means *with*) and *in* (means *in*). Relevantly, narrative texts also exhibited more frequent use of cardinal numbers compared to argumentative texts. These numerals often served to quantify and specify elements within the narrative, a tendency reinforced by prompts like *An Unforgettable Experience*, where time references (e.g., *bis 9 Uhr* (means *until 9 o’clock*)) were common. Learners also employed various verb forms (present perfect, simple present, and past simple tense) to narrate events or describe characters. By contrast, in argumentative essays, learners heavily relied on the simple present to state opinions, resulting in a lower value of Morphological Complexity Index of verbs (MCI-Verb) compared to narratives. This aligns with [16] which observed greater MCI-Verb in narratives among intermediate English learners. Additionally, argumentative essays were shorter than narratives, a finding consistent with [1], which attributed this to the more time learners spent planning argumentative essays due to the cognitive demands of structuring arguments, which reduced actual writing time.

In argumentative essays, learners frequently used “mental state and speech act verbs that characterize a person’s attitude toward a proposition” [13, p. 187], like *glauben* (means *believe*) and *denken* (means *think*), to articulate opinions or construct logical arguments. Additionally, they used topic-specific verbs like *verbessern* (means *improve*) and *entwickeln* (means *develop*) to discuss the consequences or reasons for actions, such as bringing mobile phones into classrooms or choosing between further studies and entering the workforce after graduation. These verbs typically require complement clauses [41] or accusative objects, leading to more accusatives, dependent clauses, and connectors like *wenn* (means *when*), *deshalb* (means *therefore*), and *außerdem* (means *moreover*). Although this frequent use of words essential for argumentation limited the variety of vocabulary and resulted in lower lexical richness, argumentative essays exhibited higher lexical sophistication, as measured by the mean age of active use of words, compared to narrative essays. This higher mean age of active use suggests that, while potentially using a more focused set of words, learners were employing vocabulary typically associated with more advanced stages of language acquisition. This includes abstract nouns related to topics such as *Abschluss* (means *graduation*), *Karriere* (means *career*), and *Fachrichtung* (means *major*), mirroring findings by [14] who observed a greater prevalence of abstract nouns and longer words in argumentative essays, as opposed to narratives, produced by intermediate Chinese learners of English. This pattern also partially aligns with [9]. Bi observed that elementary and intermediate Chinese L2 English learners produced greater mean length of clauses (MLC), mean length of T-units (MLT), mean length of sentences (MLS), clauses per T-unit (C/T), complex nominals per clause (CN/C), and lexical density in argumentative texts compared to narratives, although their lexical diversity was lower in argumentative texts. Similarly, our study observed higher coordinate phrases per T-unit (CP/T), complex nominals per T-unit (CN/T) and prepositional phrases per T-unit (PP/T), indicating the higher demand for information density in argumentative writing [42]. The frequent occurrence of coordinate phrases was linked to the requirements to connect ideas, to add supporting details, and to build cohesion [43]. In addition to using coordinate phrases, learners also strengthened cohesion by repeating key lemmas like *Handy* (means *mobile phone*), thereby reinforcing the central theme. In line with studies on intermediate English learners [9,14,15], our findings indicated that elementary-intermediate German learners produced greater lexical density and sophistication, more complex phrases, coordinate phrases, and dependent clauses in argumentative essays than in narratives.

Notably, our study found more connectors in argumentative essays, in contrast to [44,45], who reported more connectors in narratives. This difference could stem from proficiency levels, as our learners were elementary-intermediate, while Abdi Tabari and colleagues focused on advanced (C1) learners. Additionally, the shorter length of essays in our study may have reduced the need for connectors in narratives, and argumentative essays inherently require a higher use of connectors to ensure logical coherence throughout the text.

Overall, our findings suggest that genre exerts varying influences on different facets of linguistic complexity, with neither genre consistently outperforming the other across all facets. Our results challenge earlier studies that suggested argumentative writing consistently fosters greater linguistic complexity [1,7,46,47], and align with recent findings by [15]. In addition to the analysis above, learners demonstrated some genre awareness, but their ability to fully meet the specific linguistic demands of each genre was limited [15], particularly at the elementary-intermediate proficiency stage. As a result, their linguistic development remained uneven.

### 5.2 Linguistic complexity development in different genres

The second research question explored how multifaceted linguistic complexity develops across genres over time. The findings revealed both shared and genre-specific features in L2 German over one academic year, highlighting the importance of considering genre as a central variable in analyses of L2 writing development.

A shared characteristic across both genres was the nonlinear trajectory in the development of multiple facets of linguistic complexity among elementary-intermediate learners. This pattern was consistently observed in both narrative and argumentative writing. Such nonlinearity aligns with earlier findings [e.g., 12,15,48], which have shown that complexity indicators often fluctuate rather than develop uniformly over time. For instance, similar to [15], the present study also found nonlinear change in dependent clauses per T-unit (DC/T) across both genres. The U-shaped trajectory observed in coordinate phrases per T-unit (CP/T) within L2 German narrative writing provides a compelling example of such nonlinearity. The initial decrease from S1 to S2, followed by a rebound in S3, illustrated a dynamic developmental process that resists simple linear modeling and instead suggests constant interaction and reorganization of the learner’s linguistic system. These fluctuations can be interpreted as manifestations of the dynamic nature of L2 acquisition. Instead of following a linear, cumulative path, learners appeared to engage in periodic adjustments in how linguistic resources are deployed and integrated [49], which could lead to observable declines or periods of stability in specific complexity indicators. Our results underscore the need to model multifaceted linguistic complexity in L2 writing as a dynamic, interdependent system, with this study taking a preliminary step by identifying fluctuation patterns in complexity indicators that may inform future models of developmental dynamics in L2 German narrative and argumentative writing.

When narrowing the focus to the overlapping observation period (S2 to S3) shared by both genres – the period that allows for direct cross-genre comparisons of longitudinal development despite the staggered data collection timeline (see 3.1) – another cross-genre pattern emerged: a significant decline in lexical sophistication. During this comparable period, learners increasingly relied on high-frequency words that are actively used by native speakers at a young age. This notable trend, marked by the largest effect sizes among all indicators in both genres, contrasts with studies that noted improvements in lexical sophistication [15] or no significant change over time [1,50]. At the same time, however, other indicators of linguistic complexity exhibited clear signs of development in both genres. In narrative writing, coordinate phrases per T-unit (CP/T) showed the most substantial increase among all three indicators during this period, indicating a marked growth in syntactic coordination. Meanwhile, learners’ argumentative writing revealed significant improvements across multiple facets of complexity, including text length, morphological inflections, lexical richness, syntactic complexity and cohesive devices. A detailed discussion of these genre-specific developments is presented in the following paragraphs. Taken together, this combination of findings could be interpreted as evidence of a “complexity trade-off”, in which “the relationship between different dimensions and layers of complexity can be both supportive and competitive [...], and their correlation can change over time” [51, p. 148]. Specifically, L2 acquisition and development are assumed to require cognitive (e.g., attention, working memory) and environmental (e.g., input, learning materials) resources, which are inherently limited. Such limitations may induce competition among linguistic subsystems, compelling learners to allocate their resources strategically during language production. Consequently, learners’ focus on one complexity facet typically diminishes resources available for others – though this competitive relationship may evolve into mutual support once certain developmental levels are reached [52,53]. Empirical research [54,55] has consistently demonstrated that competition between lexical complexity and other facets, such as syntactic complexity, is particularly pronounced at lower proficiency levels. Correspondingly, our study found that in both genres, elementary-intermediate German learners appeared to prioritize the expansion of other complexity measures over the use of more sophisticated vocabulary, likely as a strategy to manage cognitive load. Similar trade-offs have been reported in previous research [53,55].

While both genres exhibited shared developmental patterns, genre-specific differences also became apparent. During the shared observation period, developmental changes in argumentative writing were more broadly distributed across linguistic complexity facets. In contrast, narrative writing showed significant change in only a limited number of indicators. This relative imbalance could be explained by the different timing and intensity of genre-specific instructional focus. In the present study, participants began practicing narrative writing in German from the first semester, as is typically the case for students without prior German experience. Consequently, it is possible that much of the complexity development in narrative writing occurred prior to the overlapping observation period, by which time narrative writing may have become a relatively familiar task. In contrast, argumentative writing was introduced later in the curriculum, and the overlapping observation period coincided with its initial phase of instructional emphasis and active learner engagement. This likely contributed to the more extensive development observed in argumentative writing during this stage.

More specifically, from S2 to S3, learners’ argumentative writing showed clear morphological, lexical, syntactic, and cohesive complexity development – some of which also continued into the following semester. Morphologically, learners increased their use of genitive and dative cases, while reducing reliance on the nominative. The dative case, in particular, exhibited a linear upward trajectory. This progression follows the typical German case acquisition sequence, which usually starts with the nominative case, followed by the gradual differentiation of the other cases [56]. This sequence also aligns with standard German language textbooks in China. Moreover, learners demonstrated linear improvement in the corrected type-token ratio (CTTR) and mean length of clauses (MLC), along with stage-wise growth in complex nominals per T-unit (CN/T). These trends are broadly consistent with findings in [10,11]. Syntactically, learners increased their use of relative pronouns and prepositional phrases in argumentative writing, while decreasing use of cardinal numbers and adverbs. These trends largely align with [12], except for differences in adverb and prepositional phrase usage, where no significant development was observed among American L2 German learners. This difference could be explained by genre effects, which Vyatkina and colleagues did not consider. Furthermore, the decline in adverb usage is unsurprising, as German learners tended to underuse adverbs compared to native speakers [57]. Regarding cohesion, argumentative writing showed a significant linear increase in global lemma overlap, paralleling the findings of [18], who reported an increase of local lemma overlap in L2 German narrative writing. One possible explanation of this difference is that argumentative texts might primarily maintain cohesion by repeating lemmas related to central themes throughout the text, whereas narratives may rely more on lemma repetition between adjacent sentences to ensure storyline continuity.

## 6 Conclusion

This study investigated the development of multifaceted linguistic complexity (morphological, lexical, syntactic and cohesive) in elementary-intermediate Chinese learners of German, focusing on genre differences (narrative vs. argumentative writing) and longitudinal developments. The findings revealed that genre effects were significant yet nuanced, challenging the assumption that one genre is consistently more complex than another. Furthermore, linguistic complexity development showed both shared and genre-specific features. In argumentative writing, complexity followed three patterns from the second to the fourth semester: continuous linear, nonlinear stage-wise, and cumulative development. In narrative writing, from the first to the third semester, complexity showed bidirectional fluctuation and nonlinear stage-wise development. Both genres demonstrated nonlinear development, underscoring the dynamic nature of second language acquisition. Additionally, learners exhibited a “complexity trade-off”, whereby efforts to improve certain linguistic complexity, such as syntactic complexity, limited the use of advanced vocabulary. This significant decline in lexical sophistication across genres contrasts with previous findings of either improvement or stability in lexical sophistication.

Despite these insights, the study was subject to several inherent methodological limitations. Although both genres were written by the same group of students and tracked across an entire academic year, the data collection periods for the two genres were staggered due to students’ proficiency limitations and the curriculum’s progression. Data for narrative writing were collected at the end of the first, second, and third semesters, while data for argumentative writing were collected at the end of the second, third, and fourth semesters. This temporal discrepancy in data collection prevented a direct comparison of the complete developmental trajectories across genres. However, the overlapping six-month period provided valuable comparative insights. Future research could extend the observation period to fully track both genres or adopt more flexible data collection methods to encourage students’ production of various genres. Additionally, the absence of standardized proficiency testing, due to practical constraints, required reliance on estimated study hours to approximate language proficiency, which could introduce imprecision in proficiency grouping and limit comparability with studies using standardized tests. Moreover, homogeneity in participants’ educational and linguistic profiles likely controlled for certain confounding variables. Nonetheless, incorporating individual-level factors may yield deeper insights into learners’ complexity development. Future research could benefit from a qualitative exploration of the observed decline in lexical sophistication, which could further illuminate how learners balance different linguistic complexity facets.

Theoretically, this study addressed an underexamined area by focusing on lower-proficiency learners, a group often underrepresented in previous L2 English research. Furthermore, unlike previous longitudinal studies that alternated between different genres at varying time points, our approach required students to write both narrative and argumentative essays at each time point during the overlapping observation period, allowing for a more direct comparison of genre effects over time. Importantly, this study also highlighted the significance of morphological complexity, a facet that has received limited attention, thus broadening the scope of linguistic complexity research beyond syntactic and lexical facets. Finally, by examining L2 German, this study contributed to the broader field of SLA research and provided empirical evidence that supports cross-linguistic comparisons.

In pedagogy, this study offers insights that extend beyond L2 German to broader second language teaching. Since no genre consistently showed greater complexity across all dimensions, educators should incorporate diverse genres into curricula to develop multifaceted linguistic competence. Educators could strategically leverage different genre effects on linguistic complexity: narrative writing for morphology, and argumentative writing for lexical, syntactic, and cohesive complexities, providing tailored support based on learners’ needs at different stages. Additionally, explicit instruction in genre awareness – helping both educators and learners recognize the distinctive linguistic features of different genres – could enhance the effectiveness of genre-based pedagogy. The observed nonlinear developmental trajectories highlight the importance of adjusting expectations, as declines in certain complexity indicators do not signify regression in ability. Finally, a multifaceted assessment framework of linguistic complexity is essential for more comprehensively tracking development and addressing gaps, ultimately fostering balanced linguistic development.

## Supporting information

S1 Table Overview of all initially considered indicators in this study. (DOCX)

S2 Table Friedman test and Wilcoxon signed-rank tests results for linguistic complexity development in L2 German argumentative writing. (DOCX)

S3 Table Friedman test and Wilcoxon signed-rank tests results for linguistic complexity development in L2 German narrative writing. (DOCX)
