# Supplementary material for: The effects of genres on the development of multifaceted linguistic complexity in Chinese learners of German: A longitudinal corpus analysis
Source: PLoS One. 2025 Jun 16;20(6):e0326250. doi: 10.1371/journal.pone.0326250 (PMC12169520; doi:10.1371/journal.pone.0326250)
Supplement: S1 Table — (DOCX) [file pone.0326250.s001.docx]

**S1 Table. Overview of all initially considered indicators in this study**

| Indicator | Category | Description | Retained |
| --- | --- | --- | --- |
| *Global complexity* |  |  |  |
| Text length |  | # of tokens | √ |
| *Morphological complexity* |  |  |  |
| MCI-Verb |  | MCI for verbs with 2 partitions, each with 5 exponents | √ |
| Inflection (nominative) |  | # of nominative case markings / # of tokens | √ |
| Inflection (genitive) |  | # of genitive case markings / # of tokens | √ |
| Inflection (dative) |  | # of dative case markings / # of tokens | √ |
| Inflection (accusative) |  | # of accusative case markings / # of tokens | √ |
| *Lexical complexity* |  |  |  |
| Lexical richness (CTTR) | Lexical Richness | # of unique word types / √(2 × # of tokens) | √ |
| Lexical density | Lexical Density | # of lexical words / # of tokens | √ |
| Word frequency | Lexical Sophistication | Σ (frequencies of all words found in *Google Books 2000*) / # of words found in *Google Books 2000* | √ |
| Mean age of active use | Lexical Sophistication | Σ (age of all writers in KCT* using words) / # of words found in KCT | √ |
| *Syntactic complexity* |  |  |  |
| Mean length of clause  (MLC) | Length of production unit | # of words / # of clauses | √ |
| Mean length of sentence  (MLS) | Length of production unit | # of words / # of sentences | × |
| Mean length of T-unit  (MLT) | Length of production unit | # of words / # of T-units | × |
| Sentence coordination  ratio (T/S) | Coordination | # of T-units / # of sentences | √ |
| Coordinate phrases per  clause (CP/C) | Coordination | # of coordinate phrases / # of clauses | × |
| Coordinate phrases per  T-unit (CP/T) | Coordination | # of coordinate phrases / # of T-units | √ |
| Sentence complexity  ratio (C/S) | Sentence complexity | # of clauses / # of sentences | √ |
| T-unit complexity ratio  (C/T) | Subordination | # of clauses / # of T-units | × |
| Complex T-unit ratio  (CT/T) | Subordination | # of complex T-units / # of T-units | × |
| Dependent clause ratio  (DC/C) | Subordination | # of dependent clauses / # of clauses | × |
| Dependent clauses per  T-unit (DC/T) | Subordination | # of dependent clauses / # of T-units | √ |
| Complex nominals per  clause (CN/C) | Particular structures | # of complex nominals / # of clauses | × |
| Complex nominals per  T-unit (CN/T) | Particular structures | # of complex nominals / # of T-units | √ |
| Verb phrases per T-unit  (VP/T) | Particular structures | # of verb phrases / # of T-units | × |
| Subordinating  conjunction density | Syntactic modifier | # of subordinating conjunctions / # of tokens | √ |
| Relative pronoun density | Syntactic modifier | # of relative pronouns / # of tokens | √ |
| Adjective density | Syntactic modifier | # of adjectives / # of tokens | √ |
| Cardinal number density | Syntactic modifier | # of cardinal numbers / # of tokens | √ |
| Adverb density | Syntactic modifier | # of adverbs / # of tokens | √ |
| Prepositional phrases per  T-unit | Syntactic modifier | # of prepositional phrases / # of T-units | √ |
| *Cohesive complexity* |  |  |  |
| All connectors per token | Local cohesion | # of all connectors / # of tokens | √ |
| Temporal connectors per  token | Local cohesion | # of temporal connectors / # of tokens | × |
| Local noun overlap | Local cohesion | # of nouns overlapping between adjacent sentences / # of sentences | × |
| Local argument overlap | Local cohesion | # of arguments overlapping between adjacent sentences / # of sentences | √ |
| Local content word  overlap | Local cohesion | # of content words overlapping between adjacent sentences / # of sentences | × |
| Local lemma overlap | Local cohesion | # of lemmas overlapping between adjacent sentences / # of sentences | × |
| Global noun overlap | Overall cohesion | # of nouns overlapping between any two sentences / # of sentences | × |
| Global argument overlap | Overall cohesion | # of arguments overlapping between any two sentences / # of sentences | √ |
| Global content word  overlap | Overall cohesion | # of content words overlapping between any two sentences / # of sentences | × |
| Global lemma overlap | Overall cohesion | # of lemmas overlapping between any two sentences / # of sentences | √ |
| **Notes.* KCT = Karlsruhe Children’s Text Corpus | | | |
