# Supplementary material for: The effects of genres on the development of multifaceted linguistic complexity in Chinese learners of German: A longitudinal corpus analysis
Source: PLoS One. 2025 Jun 16;20(6):e0326250. doi: 10.1371/journal.pone.0326250 (PMC12169520; doi:10.1371/journal.pone.0326250)
Supplement: S2 Table — (DOCX) [file pone.0326250.s002.docx]

**S2 Table Friedman test and Wilcoxon signed-rank tests results for linguistic complexity development in L2 German argumentative writing**

| Indicator | Complexity | S2 Med (Q1 - Q3) | S3 Med (Q1 - Q3) | S4 Med (Q1 - Q3) | Friedman test | | Wilcoxon test (S2 vs. S3) | | Wilcoxon test (S3 vs. S4) | | Wilcoxon test (S2 vs. S4) | | Trajectory (S2-S3-S4, S2-S4) |
| --- | --- | --- | --- | --- | --- | --- | --- | --- | --- | --- | --- | --- | --- |
|  |  |  |  |  | *χ²(df=2)* | *p*(BH) | *Z* | *p*(BH) | *Z* | *p*(BH) | *Z* | *p*(BH) |  |
| Text length | Global | 100.000 (91.500 - 124.500) | 125.000 (109.000 - 156.500) | 149.000 (140.500 - 182.500) | 29.238 | < 0.001 | -3.772 | < 0.001 | -3.303 | 0.001 | -3.980 | < 0.001 | ↑↑ / ↑ |
| MCI-Verb | Morphological | 3.000 (2.500 - 4.000) | 2.500 (1.250 - 3.000) | 2.500 (2.500 - 3.750) | 5.013 | 0.098 |  |  |  |  |  |  |  |
| Inflection (nominative) | Morphological | 0.642 (0.611 - 0.662) | 0.566 (0.514 - 0.607) | 0.550 (0.509 - 0.579) | 19.143 | < 0.001 | -3.319 | 0.001 | -1.199 | 0.273 | -3.910 | < 0.001 | ↓→ / ↓ |
| Inflection (genitive) | Morphological | 0.014 (0.000 - 0.027) | 0.037 (0.026 - 0.052) | 0.038 (0.021 - 0.045) | 18.952 | < 0.001 | -3.632 | < 0.001 | -1.512 | 0.158 | -3.041 | 0.003 | ↑→ / ↑ |
| Inflection (dative) | Morphological | 0.148 (0.112 - 0.178) | 0.214 (0.158 - 0.232) | 0.262 (0.224 - 0.294) | 18.952 | < 0.001 | -2.833 | 0.007 | -3.597 | < 0.001 | -3.771 | < 0.001 | ↑↑ / ↑ |
| Inflection (accusative) | Morphological | 0.200 (0.146 - 0.233) | 0.239 (0.193 - 0.260) | 0.198 (0.175 - 0.221) | 7.238 | 0.039 | -1.894 | 0.080 | -2.728 | 0.009 | -0.574 | 0.608 | →↓ / → |
| Lexical richness (CTTR) | Lexical | 4.861 (4.419 - 5.368) | 5.181 (4.945 - 5.363) | 5.619 (5.090 - 6.159) | 17.238 | < 0.001 | -2.868 | 0.006 | -2.172 | 0.042 | -3.736 | < 0.001 | ↑↑ / ↑ |
| Lexical density | Lexical | 0.520 (0.481 - 0.534) | 0.513 (0.496 - 0.557) | 0.521 (0.502 - 0.533) | 1.143 | 0.620 |  |  |  |  |  |  |  |
| Word frequency | Lexical | 3130.027 (2846.913 - 3614.181) | 4906.606 (4263.315 - 5798.280) | 4396.128 (3887.188 - 5682.758) | 16.095 | 0.001 | -3.771 | < 0.001 | -0.643 | 0.571 | -3.528 | < 0.001 | ↑→ / ↑ |
| Mean age of active use | Lexical | 11.115 (10.964 - 11.169) | 10.859 (10.799 - 10.945) | 10.933 (10.824 - 10.990) | 17.238 | < 0.001 | -3.806 | < 0.001 | -1.616 | 0.133 | -3.146 | 0.002 | ↓→ / ↓ |
| Mean length of clause | Syntactic | 7.167 (6.736 - 8.523) | 8.563 (7.426 - 9.087) | 9.400 (8.055 - 10.546) | 18.667 | < 0.001 | -2.520 | 0.018 | -2.416 | 0.023 | -3.180 | 0.002 | ↑↑ / ↑ |
| Sentence coordination ratio | Syntactic | 1.000 (1.000 - 1.000) | 0.909 (0.861 - 1.000) | 0.933 (0.826 - 1.026) | 5.507 | 0.077 |  |  |  |  |  |  |  |
| Coordinate phrases per T-unit | Syntactic | 0.100 (0.000 - 0.236) | 0.250 (0.143 - 0.286) | 0.100 (0.000 - 0.168) | 5.636 | 0.077 |  |  |  |  |  |  |  |
| Sentence complexity ratio | Syntactic | 1.364 (1.218 - 1.500) | 1.500 (1.226 - 1.663) | 1.364 (1.275 - 1.667) | 2.049 | 0.401 |  |  |  |  |  |  |  |
| Dependent clauses per T-unit | Syntactic | 0.333 (0.226 - 0.442) | 0.429 (0.278 - 0.646) | 0.556 (0.446 - 0.788) | 12.400 | 0.003 | -1.730 | 0.112 | -2.312 | 0.029 | -2.897 | 0.005 | →↑ / ↑ |
| Complex nominals per T-unit | Syntactic | 0.400 (0.273 - 0.550) | 0.556 (0.464 - 0.823) | 0.750 (0.567 - 0.908) | 14.857 | 0.001 | -2.763 | 0.008 | -1.147 | 0.287 | -3.528 | < 0.001 | ↑→ / ↑ |
| Subordinating conjunction density | Syntactic | 0.030 (0.020 - 0.040) | 0.029 (0.021 - 0.045) | 0.036 (0.030 - 0.042) | 3.714 | 0.184 |  |  |  |  |  |  |  |
| Relative pronoun density | Syntactic | 0.000 (0.000 - 0.000) | 0.000 (0.000 - 0.009) | 0.005 (0.000 - 0.007) | 12.566 | 0.002 | -2.666 | 0.008 | -0.103 | 0.940 | -3.059 | 0.001 | ↑→ / ↑ |
| Adjective density | Syntactic | 0.081 (0.052 - 0.090) | 0.059 (0.039 - 0.067) | 0.049 (0.040 - 0.069) | 8.000 | 0.028 | -1.999 | 0.064 | -0.956 | 0.384 | -2.416 | 0.023 | →→ / ↓ |
| Cardinal number density | Syntactic | 0.000 (0.000 - 0.010) | 0.000 (0.000 - 0.000) | 0.000 (0.000 - 0.005) | 7.161 | 0.036 | -2.371 | 0.025 | -1.992 | 0.082 | -1.601 | 0.147 | ↓→ / → |
| Adverb density | Syntactic | 0.079 (0.050 - 0.092) | 0.046 (0.036 - 0.056) | 0.045 (0.037 - 0.052) | 13.238 | 0.002 | -2.902 | 0.005 | -0.469 | 0.670 | -3.528 | < 0.001 | ↓→ / ↓ |
| Prepositional phrases per T-unit | Syntactic | 0.563 (0.364 - 0.798) | 1.214 (0.813 - 1.333) | 1.333 (1.112 - 1.882) | 26.096 | < 0.001 | -3.771 | < 0.001 | -2.260 | 0.033 | -3.911 | < 0.001 | ↑↑ / ↑ |
| Local argument overlap | Cohesive | 0.083 (0.000 - 0.138) | 0.000 (0.000 - 0.074) | 0.000 (0.000 - 0.000) | 11.704 | 0.003 | -1.658 | 0.127 | -1.690 | 0.133 | -2.970 | 0.003 | →→ / ↓ |
| Global argument overlap | Cohesive | 0.545 (0.388 - 1.161) | 0.667 (0.464 - 0.928) | 0.818 (0.592 - 1.111) | 2.000 | 0.405 |  |  |  |  |  |  |  |
| All connectors per token | Cohesive | 0.202 (0.130 - 0.251) | 0.170 (0.089 - 0.252) | 0.117 (0.056 - 0.182) | 6.381 | 0.055 |  |  |  |  |  |  |  |
| Global lemma overlap | Cohesive | 0.111 (0.033 - 0.282) | 0.500 (0.317 - 0.818) | 0.714 (0.564 - 1.002) | 25.268 | < 0.001 | -3.582 | < 0.001 | -2.103 | 0.050 | -3.980 | < 0.001 | ↑↑ / ↑ |

*Notes*. S2, S3, and S4 refer to the second, third, and fourth semesters, respectively. Median (Med) values are shown with interquartile ranges (Q1 - Q3). *p*-values were computed using Friedman tests and Wilcoxon signed-rank tests, with Benjamini-Hochberg (BH) adjustment for multiple comparisons. Trajectory arrows indicate significant changes: ↑ (increase), ↓ (decrease), → (no significant change).
