# Supplementary material for: The effects of genres on the development of multifaceted linguistic complexity in Chinese learners of German: A longitudinal corpus analysis
Source: PLoS One. 2025 Jun 16;20(6):e0326250. doi: 10.1371/journal.pone.0326250 (PMC12169520; doi:10.1371/journal.pone.0326250)
Supplement: S3 Table — (DOCX) [file pone.0326250.s003.docx]

**S3 Table Friedman test and Wilcoxon signed-rank tests results for linguistic complexity development in L2 German narrative writing**

| Indicator | Complexity | S1 Med (Q1 - Q3) | S2 Med (Q1 - Q3) | S3 Med (Q1 - Q3) | Friedman test | | Wilcoxon test (S1 vs. S2) | | Wilcoxon test (S2 vs. S3) | | Wilcoxon test (S1 vs. S3) | | Trajectory (S1-S2-S3, S1-S3) |
| --- | --- | --- | --- | --- | --- | --- | --- | --- | --- | --- | --- | --- | --- |
|  |  |  |  |  | *χ² (df=2)* | *p*(BH) | *Z* | *p*(BH) | *Z* | *p*(BH) | *Z* | *p*(BH) |  |
| Text length | Global | 140.000 (115.000 - 158.000) | 139.000 (101.500 - 153.000) | 129.000 (109.000 - 163.000) | 4.795 | 0.134 |  |  |  |  |  |  |  |
| MCI-Verb | Morphological | 3.000 (2.500 - 3.500) | 4.000 (4.000 - 4.500) | 4.500 (4.000 - 5.000) | 13.273 | 0.003 | -3.010 | 0.003 | -1.008 | 0.405 | -3.107 | 0.002 | ↑→ / ↑ |
| Inflection (nominative) | Morphological | 0.541 (0.492 - 0.570) | 0.580 (0.530 - 0.650) | 0.591 (0.552 - 0.610) | 10.381 | 0.010 | -3.180 | 0.002 | -0.122 | 0.919 | -2.972 | 0.003 | ↑→ / ↑ |
| Inflection (genitive) | Morphological | 0.069 (0.054 - 0.095) | 0.038 (0.020 - 0.050) | 0.025 (0.019 - 0.040) | 18.667 | < 0.001 | -3.354 | 0.001 | -0.956 | 0.416 | -3.597 | < 0.001 | ↓→ / ↓ |
| Inflection (dative) | Morphological | 0.173 (0.154 - 0.210) | 0.225 (0.185 - 0.244) | 0.217 (0.200 - 0.254) | 6.000 | 0.080 |  |  |  |  |  |  |  |
| Inflection (accusative) | Morphological | 0.193 (0.178 - 0.222) | 0.139 (0.122 - 0.152) | 0.162 (0.104 - 0.191) | 12.667 | 0.004 | -3.250 | 0.002 | -0.400 | 0.755 | -2.972 | 0.003 | ↓→ / ↓ |
| Lexical richness (CTTR) | Lexical | 4.933 (4.177 - 5.229) | 5.503 (5.254 - 5.965) | 5.677 (5.225 - 5.912) | 14.386 | 0.002 | -3.389 | 0.001 | -1.195 | 0.310 | -3.597 | < 0.001 | ↑→ / ↑ |
| Lexical density | Lexical | 0.510 (0.495 - 0.529) | 0.450 (0.424 - 0.474) | 0.470 (0.448 - 0.487) | 19.143 | < 0.001 | -3.424 | 0.001 | -1.790 | 0.114 | -3.146 | 0.002 | ↓→ / ↓ |
| Word frequency | Lexical | 3384.483 (2932.826 - 3990.353) | 3354.501 (2923.009 - 3867.559) | 3780.694 (3429.351 - 4443.916) | 7.143 | 0.047 | -0.122 | 0.919 | -2.277 | 0.033 | -2.311 | 0.032 | →↑ / ↑ |
| Mean age of active use | Lexical | 10.737 (10.683 - 10.807) | 10.719 (10.657 - 10.773) | 10.594 (10.510 - 10.686) | 10.286 | 0.010 | -0.504 | 0.691 | -2.868 | 0.005 | -3.146 | 0.002 | →↓ / ↓ |
| Mean length of clause | Syntactic | 5.379 (5.147 - 5.833) | 6.577 (5.927 - 7.395) | 6.696 (6.269 - 7.526) | 21.238 | < 0.001 | -3.702 | < 0.001 | -0.921 | 0.427 | -3.945 | < 0.001 | ↑→ / ↑ |
| Sentence coordination ratio | Syntactic | 1.000 (0.956 - 1.059) | 0.923 (0.892 - 1.038) | 1.000 (0.887 - 1.000) | 1.627 | 0.551 |  |  |  |  |  |  |  |
| Coordinate phrases per T-unit | Syntactic | 0.138 (0.070 - 0.223) | 0.059 (0.000 - 0.085) | 0.105 (0.052 - 0.191) | 8.951 | 0.017 | -3.024 | 0.003 | -2.296 | 0.032 | -0.991 | 0.405 | ↓↑ / → |
| Sentence complexity ratio | Syntactic | 1.143 (1.038 - 1.326) | 1.364 (1.233 - 1.641) | 1.333 (1.300 - 1.641) | 10.927 | 0.008 | -3.146 | 0.002 | -0.604 | 0.634 | -3.285 | 0.001 | ↑→ / ↑ |
| Dependent clauses per T-unit | Syntactic | 0.000 (0.000 - 0.000) | 0.250 (0.138 - 0.376) | 0.333 (0.191 - 0.458) | 32.488 | < 0.001 | -4.015 | < 0.001 | -1.228 | 0.297 | -4.015 | < 0.001 | ↑→ / ↑ |
| Complex nominals per T-unit | Syntactic | 0.261 (0.200 - 0.286) | 0.273 (0.192 - 0.478) | 0.375 (0.216 - 0.604) | 2.952 | 0.331 |  |  |  |  |  |  |  |
| Subordinating conjunction density | Syntactic | 0.000 (0.000 - 0.000) | 0.029 (0.017 - 0.035) | 0.028 (0.019 - 0.032) | 31.524 | < 0.001 | -4.015 | < 0.001 | -0.122 | 0.919 | -4.015 | < 0.001 | ↑→ / ↑ |
| Relative pronoun density | Syntactic | 0.000 (0.000 - 0.000) | 0.000 (0.000 - 0.003) | 0.000 (0.000 - 0.000) | 1.613 | 0.564 |  |  |  |  |  |  |  |
| Adjective density | Syntactic | 0.069 (0.056 - 0.085) | 0.069 (0.059 - 0.085) | 0.071 (0.055 - 0.086) | 0.000 | 1.000 |  |  |  |  |  |  |  |
| Cardinal number density | Syntactic | 0.022 (0.016 - 0.034) | 0.006 (0.000 - 0.008) | 0.008 (0.002 - 0.013) | 18.829 | < 0.001 | -3.667 | < 0.001 | -1.771 | 0.116 | -2.798 | 0.006 | ↓→ / ↓ |
| Adverb density | Syntactic | 0.066 (0.050 - 0.091) | 0.059 (0.050 - 0.074) | 0.072 (0.053 - 0.092) | 0.381 | 0.917 |  |  |  |  |  |  |  |
| Prepositional phrases per T-unit | Syntactic | 0.316 (0.252 - 0.407) | 0.571 (0.410 - 0.789) | 0.750 (0.542 - 0.861) | 21.422 | < 0.001 | -3.667 | < 0.001 | -1.564 | 0.171 | -3.920 | < 0.001 | ↑→ / ↑ |
| Local argument overlap | Cohesive | 0.069 (0.041 - 0.150) | 0.067 (0.000 - 0.091) | 0.083 (0.000 - 0.118) | 1.872 | 0.512 |  |  |  |  |  |  |  |
| Global argument overlap | Cohesive | 1.136 (0.826 - 1.487) | 1.067 (0.550 - 1.652) | 0.895 (0.423 - 1.589) | 2.627 | 0.368 |  |  |  |  |  |  |  |
| All connectors per token | Cohesive | 0.116 (0.085 - 0.148) | 0.127 (0.080 - 0.177) | 0.109 (0.063 - 0.148) | 0.095 | 1.000 |  |  |  |  |  |  |  |
| Global lemma overlap | Cohesive | 0.192 (0.146 - 0.272) | 0.077 (0.000 - 0.150) | 0.111 (0.070 - 0.206) | 10.145 | 0.010 | -3.215 | 0.002 | -1.251 | 0.293 | -1.547 | 0.176 | ↓→ / → |

*Notes*. S1, S2, and S3 refer to the first, second, and third semesters, respectively. Median (Med) values are shown with interquartile ranges (Q1 - Q3). *p*-values were computed using Friedman tests and Wilcoxon signed-rank tests, with Benjamini-Hochberg (BH) adjustment for multiple comparisons. Trajectory arrows indicate significant changes: ↑ (increase), ↓ (decrease), → (no significant change).
